# *rac*-5-Acetyl-6-hy­droxy-3,6-dimethyl-4-phenyl-4,5,6,7-tetra­hydro-2*H*-indazole

**DOI:** 10.1107/S1600536811013195

**Published:** 2011-04-13

**Authors:** Abel M. Maharramov, Arif I. Ismiyev, Bahruz A. Rashidov, Ilkin V. Aliyev

**Affiliations:** a Baku State University, Z. Khalilov St 23, Baku, AZ-1148, Azerbaijan

## Abstract

The title compound, C_17_H_20_N_2_O_2_, is chiral but crystallizes in a centrosymmetric space group as a racemate, the relative configuration at the stereogenic centres being 2S*,3R*,4S*. The cyclo­hexane ring adopts a half-chair conformation while the pyrazole ring has an envelope conformation. The crystal packing displays inter­molecular O—H⋯N and N—H⋯O hydrogen bonding.

## Related literature

For background to the use of β-cyclo­ketols as synthons in syntheses of pyrazoles, see: Pramula *et al.* (1985 ▶).
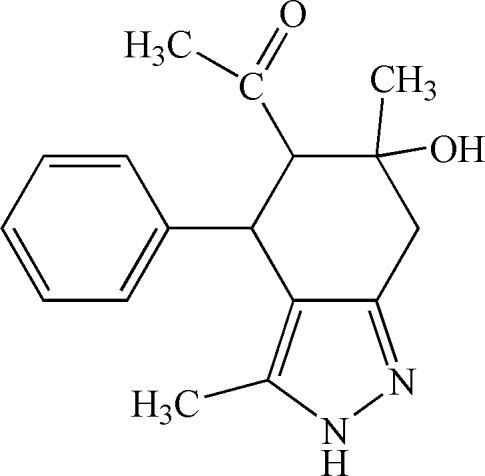


## Experimental

### 

#### Crystal data

C_17_H_20_N_2_O_2_
*M* *_r_* = 284.35
Monoclinic, 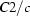

*a* = 18.6999 (9) Å
*b* = 5.6415 (3) Å
*c* = 28.4855 (14) Å
β = 94.498 (1)°
*V* = 2995.8 (3) Å^3^
*Z* = 8
Mo *K*α radiation
μ = 0.08 mm^−1^
*T* = 296 K
0.30 × 0.30 × 0.20 mm

#### Data collection

Bruker APEXII CCD area-detector diffractometer
Absorption correction: multi-scan (*SADABS*; Sheldrick, 2003 ▶) *T*_min_ = 0.975, *T*_max_ = 0.984
16597 measured reflections
3709 independent reflections
3079 reflections with *I* > 2σ(*I*)
*R*_int_ = 0.021

#### Refinement

*R*[*F* ^2^ > 2σ(*F* ^2^)] = 0.045
*wR*(*F* ^2^) = 0.129
*S* = 1.00
3709 reflections
193 parameters
H-atom parameters constrained
Δρ_max_ = 0.32 e Å^−3^
Δρ_min_ = −0.16 e Å^−3^

### 

Data collection: *APEX2* (Bruker, 2005 ▶); cell refinement: *SAINT-Plus*, (Bruker, 2001 ▶); data reduction: *SAINT-Plus*; program(s) used to solve structure: *SHELXS97* (Sheldrick, 2008 ▶); program(s) used to refine structure: *SHELXL97* (Sheldrick, 2008 ▶); molecular graphics: *SHELXTL* (Sheldrick, 2008 ▶); software used to prepare material for publication: *SHELXL97*.

## Supplementary Material

Crystal structure: contains datablocks Global, I. DOI: 10.1107/S1600536811013195/kp2321sup1.cif

Structure factors: contains datablocks I. DOI: 10.1107/S1600536811013195/kp2321Isup2.hkl

Additional supplementary materials:  crystallographic information; 3D view; checkCIF report

## Figures and Tables

**Table 1:** Hydrogen-bond geometry (Å, °)

*D*—H⋯*A*	*D*—H	H⋯*A*	*D*⋯*A*	*D*—H⋯*A*
O2—H2*A*⋯N1^i^	0.82	2.03	2.8487 (14)	178
N2—H2*C*⋯O1^ii^	0.86	2.11	2.9587 (15)	168

Symmetry codes: (i) 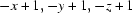; (ii) 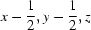.

**Fig. 1.**
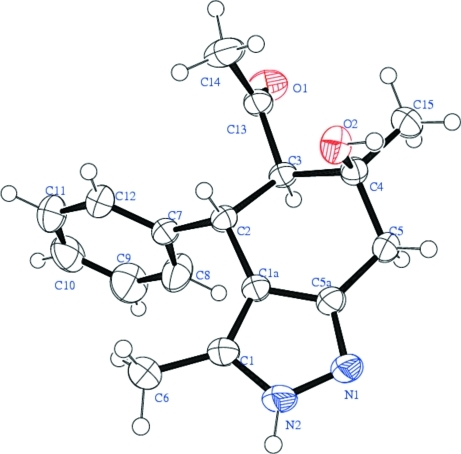
The molecular structure of the title compound with the atomic numbering scheme. Displacement ellipsoids were drawn at the 30% probability level.

**Fig. 2.**
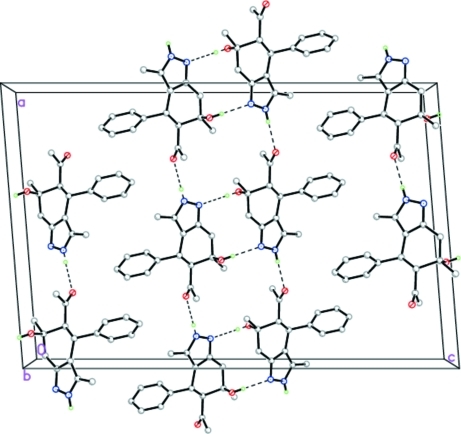
Crystal packing of (I) with hydrogen bonding (dashed lines). H atoms not involved in hydrogen bonding have been omitted for clarity.

## supplementary crystallographic information

### Comment

The exploitation of a simple molecule with different functionalities for the synthesis of heterocycles is usefula  approach. In fact, the β-cycloketols has been used as an effective synthon in some projected syntheses of pyrazoles (Pramula *et al.* 1985).

In the title compound, C_17_H_20_N_2_O_2_ (I), the cyclohexane ring adopts a half-chair conformation (Fig. 1). Cyclohexane ring has a chair conformation. The phenyl ring is in a pseudo-equatorial position. The torsion angle between the acetyl group and the phenyl substituent (C7—C2—C3—C13) is 64.88 (13) °indicating the pseudo-axial location of hydrogen atoms at C2 and C3. The crystal of (I) is racemate and consists of enantiomeric pairs where the relative configuration of the centres are 2*S**,3*R**,4*S**. The crystal structure involves N—H···O and O—H···N intermolecular hydrogen bonds (Table 1 and Fig. 2).

### Experimental

2,4-Diacetyl-5-hydroxy-5-metyl-3-phenilcyclohexanon (20 mmol), hydrazine hydrate (20 mmol) were dissolved in 20 mL ethanol. The mixture was stirred at 345–350 K for 10 h. After cooling to room temperature, white crystals were obtained. The crystals were filtered off and washed with ethanol. Then, they were dissolved in ethanol (50 mL) and recrystallised to yield colourless block-shaped crystals suitable for data collection.

### Refinement

The hydrogen atoms of the NH and OH-groups of the molecule were located in a difference-Fourier map and included in the refinement with fixed positional and isotropic displacement parameters [*U*_iso_(H) = 1.5*U*_eq_(C) for CH_3_-group and *U*_iso_(H) = 1.2*U*_eq_(N) for amino groups]. The other hydrogen atoms were placed in calculated positions and refined in the riding model with the fixed isotropic displacement parameters [*U*_iso_(H) = 1.2*U*_eq_(C)].

### Crystal data

**Table:**

C_17_H_20_N_2_O_2_	*F*(000) = 1216
*M**_r_* = 284.35	*D*_x_ = 1.261 Mg m^−^^3^
Monoclinic, *C*2/*c*	Mo *K*α radiation, λ = 0.71073 Å
Hall symbol: -C 2yc	Cell parameters from 6277 reflections
*a* = 18.6999 (9) Å	θ = 2.5–28.2°
*b* = 5.6415 (3) Å	µ = 0.08 mm^−^^1^
*c* = 28.4855 (14) Å	*T* = 296 K
β = 94.498 (1)°	Prism, colorless
*V* = 2995.8 (3) Å^3^	0.30 × 0.30 × 0.20 mm
*Z* = 8	

### Data collection

**Table:**

Bruker APEXII CCD area-detector diffractometer	3709 independent reflections
Radiation source: fine-focus sealed tube	3079 reflections with *I* > 2σ(*I*)
graphite	*R*_int_ = 0.021
phi and ω scans	θ_max_ = 28.3°, θ_min_ = 2.2°
Absorption correction: multi-scan (*SADABS*; Sheldrick, 2003)	*h* = −24→24
*T*_min_ = 0.975, *T*_max_ = 0.984	*k* = −7→7
16597 measured reflections	*l* = −38→37

### Refinement

**Table:**

Refinement on *F*^2^	Primary atom site location: structure-invariant direct methods
Least-squares matrix: full	Secondary atom site location: difference Fourier map
*R*[*F*^2^ > 2σ(*F*^2^)] = 0.045	Hydrogen site location: difference Fourier map
*wR*(*F*^2^) = 0.129	H-atom parameters constrained
*S* = 1.00	*w* = 1/[σ^2^(*F*_o_^2^) + (0.0701*P*)^2^ + 1.7992*P*] where *P* = (*F*_o_^2^ + 2*F*_c_^2^)/3
3709 reflections	(Δ/σ)_max_ = 0.001
193 parameters	Δρ_max_ = 0.32 e Å^−^^3^
0 restraints	Δρ_min_ = −0.16 e Å^−^^3^

### Special details

**Table:**

Geometry. All e.s.d.'s (except the e.s.d. in the dihedral angle between two l.s. planes) are estimated using the full covariance matrix. The cell e.s.d.'s are taken into account individually in the estimation of e.s.d.'s in distances, angles and torsion angles; correlations between e.s.d.'s in cell parameters are only used when they are defined by crystal symmetry. An approximate (isotropic) treatment of cell e.s.d.'s is used for estimating e.s.d.'s involving l.s. planes.
Refinement. Refinement of *F*^2^ against ALL reflections. The weighted *R*-factor *wR* and goodness of fit *S* are based on *F*^2^, conventional *R*-factors *R* are based on *F*, with *F* set to zero for negative *F*^2^. The threshold expression of *F*^2^ > σ(*F*^2^) is used only for calculating *R*-factors(gt) *etc*. and is not relevant to the choice of reflections for refinement. *R*-factors based on *F*^2^ are statistically about twice as large as those based on *F*, and *R*- factors based on ALL data will be even larger.

### Fractional atomic coordinates and isotropic or equivalent isotropic displacement parameters (Å^2^)

**Table:**

	*x*	*y*	*z*	*U*_iso_*/*U*_eq_	
O1	0.76048 (5)	0.7902 (2)	0.61991 (4)	0.0481 (3)	
O2	0.62374 (5)	0.43848 (16)	0.52715 (3)	0.0372 (2)	
H2A	0.6097	0.4443	0.4992	0.056*	
N1	0.42526 (6)	0.5528 (2)	0.57008 (4)	0.0369 (3)	
N2	0.41169 (6)	0.3900 (2)	0.60311 (4)	0.0390 (3)	
H2C	0.3693	0.3393	0.6074	0.047*	
C1	0.47121 (7)	0.3149 (3)	0.62867 (5)	0.0347 (3)	
C1A	0.52770 (6)	0.4380 (2)	0.61152 (4)	0.0291 (3)	
C2	0.60733 (6)	0.4279 (2)	0.62496 (4)	0.0273 (2)	
H2B	0.6259	0.2787	0.6132	0.033*	
C3	0.64412 (6)	0.6363 (2)	0.60052 (4)	0.0266 (2)	
H3A	0.6312	0.7817	0.6167	0.032*	
C4	0.61636 (6)	0.6655 (2)	0.54788 (4)	0.0284 (2)	
C5	0.53694 (7)	0.7403 (2)	0.54570 (5)	0.0335 (3)	
H5A	0.5334	0.9023	0.5567	0.040*	
H5B	0.5164	0.7336	0.5134	0.040*	
C5A	0.49626 (6)	0.5808 (2)	0.57540 (4)	0.0313 (3)	
C6	0.46830 (8)	0.1288 (3)	0.66523 (6)	0.0514 (4)	
H6A	0.4196	0.1082	0.6729	0.077*	
H6B	0.4975	0.1754	0.6929	0.077*	
H6C	0.4859	−0.0178	0.6535	0.077*	
C7	0.62509 (6)	0.4376 (2)	0.67804 (4)	0.0309 (3)	
C8	0.59834 (10)	0.6182 (3)	0.70426 (5)	0.0516 (4)	
H8A	0.5690	0.7324	0.6892	0.062*	
C9	0.61420 (11)	0.6334 (4)	0.75247 (6)	0.0605 (5)	
H9A	0.5956	0.7568	0.7694	0.073*	
C10	0.65747 (10)	0.4662 (4)	0.77516 (5)	0.0561 (4)	
H10A	0.6686	0.4763	0.8075	0.067*	
C11	0.68412 (9)	0.2846 (4)	0.74998 (6)	0.0565 (4)	
H11A	0.7131	0.1701	0.7653	0.068*	
C12	0.66811 (8)	0.2700 (3)	0.70155 (5)	0.0422 (3)	
H12A	0.6866	0.1457	0.6848	0.051*	
C13	0.72575 (6)	0.6185 (2)	0.60593 (4)	0.0324 (3)	
C14	0.76231 (8)	0.3969 (3)	0.59270 (7)	0.0492 (4)	
H14A	0.8133	0.4200	0.5960	0.074*	
H14B	0.7476	0.3575	0.5606	0.074*	
H14C	0.7496	0.2701	0.6129	0.074*	
C15	0.65806 (8)	0.8528 (3)	0.52295 (5)	0.0392 (3)	
H15A	0.6384	0.8680	0.4909	0.059*	
H15B	0.7075	0.8065	0.5234	0.059*	
H15C	0.6546	1.0021	0.5388	0.059*	

### Atomic displacement parameters (Å^2^)

**Table:**

	*U*^11^	*U*^22^	*U*^33^	*U*^12^	*U*^13^	*U*^23^
O1	0.0303 (5)	0.0525 (6)	0.0605 (7)	−0.0132 (4)	−0.0030 (4)	−0.0091 (5)
O2	0.0473 (5)	0.0326 (5)	0.0306 (4)	0.0027 (4)	−0.0034 (4)	−0.0043 (4)
N1	0.0252 (5)	0.0509 (7)	0.0342 (5)	−0.0004 (5)	−0.0011 (4)	0.0027 (5)
N2	0.0234 (5)	0.0517 (7)	0.0418 (6)	−0.0053 (5)	0.0024 (4)	0.0039 (5)
C1	0.0266 (6)	0.0406 (7)	0.0372 (6)	−0.0024 (5)	0.0034 (5)	0.0025 (5)
C1A	0.0241 (5)	0.0327 (6)	0.0301 (6)	−0.0016 (4)	0.0005 (4)	0.0015 (5)
C2	0.0237 (5)	0.0292 (6)	0.0286 (5)	0.0000 (4)	−0.0005 (4)	0.0015 (4)
C3	0.0220 (5)	0.0275 (5)	0.0299 (5)	−0.0015 (4)	0.0004 (4)	−0.0016 (4)
C4	0.0277 (5)	0.0289 (6)	0.0284 (5)	−0.0011 (4)	0.0008 (4)	0.0005 (4)
C5	0.0293 (6)	0.0365 (6)	0.0338 (6)	0.0014 (5)	−0.0019 (5)	0.0066 (5)
C5A	0.0256 (6)	0.0375 (6)	0.0302 (6)	−0.0003 (5)	−0.0009 (4)	0.0005 (5)
C6	0.0388 (8)	0.0555 (9)	0.0611 (10)	−0.0003 (7)	0.0110 (7)	0.0223 (8)
C7	0.0254 (5)	0.0364 (6)	0.0305 (6)	−0.0010 (5)	−0.0006 (4)	0.0036 (5)
C8	0.0628 (10)	0.0550 (9)	0.0363 (7)	0.0217 (8)	−0.0006 (7)	−0.0010 (7)
C9	0.0754 (12)	0.0694 (12)	0.0368 (8)	0.0071 (10)	0.0062 (8)	−0.0096 (8)
C10	0.0557 (9)	0.0811 (13)	0.0306 (7)	−0.0116 (9)	−0.0017 (6)	0.0079 (8)
C11	0.0501 (9)	0.0747 (12)	0.0429 (8)	0.0074 (8)	−0.0067 (7)	0.0210 (8)
C12	0.0390 (7)	0.0463 (8)	0.0406 (7)	0.0067 (6)	0.0000 (6)	0.0088 (6)
C13	0.0245 (5)	0.0397 (7)	0.0326 (6)	−0.0046 (5)	0.0002 (4)	0.0021 (5)
C14	0.0283 (6)	0.0477 (8)	0.0723 (10)	0.0031 (6)	0.0074 (6)	−0.0021 (8)
C15	0.0376 (7)	0.0406 (7)	0.0402 (7)	−0.0055 (6)	0.0076 (5)	0.0066 (6)

### Geometric parameters (Å, °)

**Table:**

O1—C13	1.2157 (17)	C6—H6A	0.9600
O2—C4	1.4215 (15)	C6—H6B	0.9600
O2—H2A	0.8200	C6—H6C	0.9600
N1—C5A	1.3338 (16)	C7—C8	1.380 (2)
N1—N2	1.3529 (16)	C7—C12	1.3803 (18)
N2—C1	1.3496 (17)	C8—C9	1.385 (2)
N2—H2C	0.8600	C8—H8A	0.9300
C1—C1A	1.3847 (17)	C9—C10	1.371 (3)
C1—C6	1.483 (2)	C9—H9A	0.9300
C1A—C5A	1.3996 (17)	C10—C11	1.367 (3)
C1A—C2	1.5093 (16)	C10—H10A	0.9300
C2—C7	1.5235 (16)	C11—C12	1.391 (2)
C2—C3	1.5543 (16)	C11—H11A	0.9300
C2—H2B	0.9800	C12—H12A	0.9300
C3—C13	1.5256 (16)	C13—C14	1.488 (2)
C3—C4	1.5566 (16)	C14—H14A	0.9600
C3—H3A	0.9800	C14—H14B	0.9600
C4—C15	1.5218 (17)	C14—H14C	0.9600
C4—C5	1.5405 (17)	C15—H15A	0.9600
C5—C5A	1.4847 (18)	C15—H15B	0.9600
C5—H5A	0.9700	C15—H15C	0.9600
C5—H5B	0.9700		
			
C4—O2—H2A	109.5	C1—C6—H6B	109.5
C5A—N1—N2	103.94 (10)	H6A—C6—H6B	109.5
C1—N2—N1	113.36 (11)	C1—C6—H6C	109.5
C1—N2—H2C	123.3	H6A—C6—H6C	109.5
N1—N2—H2C	123.3	H6B—C6—H6C	109.5
N2—C1—C1A	105.78 (12)	C8—C7—C12	117.74 (12)
N2—C1—C6	121.79 (12)	C8—C7—C2	120.25 (11)
C1A—C1—C6	132.37 (12)	C12—C7—C2	122.01 (12)
C1—C1A—C5A	105.07 (11)	C7—C8—C9	121.61 (15)
C1—C1A—C2	131.02 (11)	C7—C8—H8A	119.2
C5A—C1A—C2	123.88 (11)	C9—C8—H8A	119.2
C1A—C2—C7	112.60 (10)	C10—C9—C8	119.82 (17)
C1A—C2—C3	108.68 (9)	C10—C9—H9A	120.1
C7—C2—C3	110.35 (9)	C8—C9—H9A	120.1
C1A—C2—H2B	108.4	C11—C10—C9	119.62 (15)
C7—C2—H2B	108.4	C11—C10—H10A	120.2
C3—C2—H2B	108.4	C9—C10—H10A	120.2
C13—C3—C2	112.29 (10)	C10—C11—C12	120.39 (15)
C13—C3—C4	111.09 (9)	C10—C11—H11A	119.8
C2—C3—C4	112.68 (9)	C12—C11—H11A	119.8
C13—C3—H3A	106.8	C7—C12—C11	120.82 (15)
C2—C3—H3A	106.8	C7—C12—H12A	119.6
C4—C3—H3A	106.8	C11—C12—H12A	119.6
O2—C4—C15	111.21 (10)	O1—C13—C14	120.56 (12)
O2—C4—C5	110.78 (10)	O1—C13—C3	119.02 (12)
C15—C4—C5	108.59 (10)	C14—C13—C3	120.39 (11)
O2—C4—C3	105.60 (9)	C13—C14—H14A	109.5
C15—C4—C3	112.25 (10)	C13—C14—H14B	109.5
C5—C4—C3	108.38 (9)	H14A—C14—H14B	109.5
C5A—C5—C4	110.28 (10)	C13—C14—H14C	109.5
C5A—C5—H5A	109.6	H14A—C14—H14C	109.5
C4—C5—H5A	109.6	H14B—C14—H14C	109.5
C5A—C5—H5B	109.6	C4—C15—H15A	109.5
C4—C5—H5B	109.6	C4—C15—H15B	109.5
H5A—C5—H5B	108.1	H15A—C15—H15B	109.5
N1—C5A—C1A	111.85 (11)	C4—C15—H15C	109.5
N1—C5A—C5	123.86 (11)	H15A—C15—H15C	109.5
C1A—C5A—C5	124.28 (11)	H15B—C15—H15C	109.5
C1—C6—H6A	109.5		
			
C5A—N1—N2—C1	−0.17 (16)	N2—N1—C5A—C1A	−0.17 (15)
N1—N2—C1—C1A	0.43 (16)	N2—N1—C5A—C5	178.45 (12)
N1—N2—C1—C6	−177.22 (13)	C1—C1A—C5A—N1	0.43 (15)
N2—C1—C1A—C5A	−0.50 (15)	C2—C1A—C5A—N1	178.64 (11)
C6—C1—C1A—C5A	176.80 (16)	C1—C1A—C5A—C5	−178.19 (12)
N2—C1—C1A—C2	−178.53 (13)	C2—C1A—C5A—C5	0.0 (2)
C6—C1—C1A—C2	−1.2 (3)	C4—C5—C5A—N1	−158.94 (12)
C1—C1A—C2—C7	−47.39 (18)	C4—C5—C5A—C1A	19.52 (18)
C5A—C1A—C2—C7	134.90 (13)	C1A—C2—C7—C8	−53.32 (17)
C1—C1A—C2—C3	−169.96 (13)	C3—C2—C7—C8	68.31 (16)
C5A—C1A—C2—C3	12.33 (16)	C1A—C2—C7—C12	127.01 (13)
C1A—C2—C3—C13	−171.20 (10)	C3—C2—C7—C12	−111.37 (13)
C7—C2—C3—C13	64.88 (13)	C12—C7—C8—C9	0.5 (3)
C1A—C2—C3—C4	−44.84 (13)	C2—C7—C8—C9	−179.23 (16)
C7—C2—C3—C4	−168.76 (9)	C7—C8—C9—C10	0.0 (3)
C13—C3—C4—O2	74.30 (12)	C8—C9—C10—C11	−0.5 (3)
C2—C3—C4—O2	−52.70 (12)	C9—C10—C11—C12	0.6 (3)
C13—C3—C4—C15	−47.03 (14)	C8—C7—C12—C11	−0.4 (2)
C2—C3—C4—C15	−174.04 (10)	C2—C7—C12—C11	179.28 (13)
C13—C3—C4—C5	−166.96 (10)	C10—C11—C12—C7	−0.1 (3)
C2—C3—C4—C5	66.04 (12)	C2—C3—C13—O1	−129.62 (13)
O2—C4—C5—C5A	65.66 (13)	C4—C3—C13—O1	103.16 (14)
C15—C4—C5—C5A	−171.94 (11)	C2—C3—C13—C14	52.50 (16)
C3—C4—C5—C5A	−49.75 (13)	C4—C3—C13—C14	−74.72 (15)

### Hydrogen-bond geometry (Å, °)

**Table:**

*D*—H···*A*	*D*—H	H···*A*	*D*···*A*	*D*—H···*A*
O2—H2A···N1^i^	0.82	2.03	2.8487 (14)	178
N2—H2C···O1^ii^	0.86	2.11	2.9587 (15)	168

Symmetry codes: (i) −*x*+1, −*y*+1, −*z*+1; (ii) *x*−1/2, *y*−1/2, *z*.
